# Acinar cell carcinoma: a report of 19 cases with a brief review of the literature

**DOI:** 10.1186/s12957-016-0919-0

**Published:** 2016-06-28

**Authors:** Yu Wang, Sinan Wang, Xuan Zhou, Hongyuan Zhou, Yunlong Cui, Qiang Li, Lun Zhang

**Affiliations:** The Department of Maxillofacial and Otorhinolaryngology Oncology, Tianjin Medical University Cancer Institute & Hospital, Key Laboratory of Cancer Prevention and Therapy, Tianjin Cancer Institute, National Clinical Research Center of Cancer, Tianjin, 300060 China; Department of Gastroenterology and Hepatology, General Hospital, Tianjin Medical University, Tianjin, China; Department of Hepatobiliary Surgery, Tianjin Medical University Cancer Institute & Hospital, Key Laboratory of Cancer Prevention and Therapy, Tianjin Cancer Institute, National Clinical Research Center of Cancer, Tianjin, 300060 China

**Keywords:** Pancreatic cancer, Acinar cell carcinoma, Resection, Surgery, Pancreatectomy, Pancreas

## Abstract

**Background:**

Acinar cell carcinoma (ACC) is a relatively rare pancreatic neoplasm with poorly defined prognosis. This study aimed to investigate this rare pancreatic neoplasm through comparing patients with ACC to pancreatic ductal cell adenocarcinoma (DCA).

**Methods:**

Tianjin Medical University Cancer Institute and Hospital pathology database was reviewed from 1995 to 2015, and 19 patients with pathologically confirmed ACC were enrolled while 19 conventional DCA patients assigned randomly as control. Retrospective review and follow-up were performed for each patient. Regression methods were used to identify differences between ACC and DCA.

**Results:**

In our study, most patients suffered from abdominal or back pain, and no lipase hypersecretion syndrome was observed. For ACC, resected cases had better survival than those without resection, and earlier staging was related to longer survival. Resection with postoperative adjuvant therapy had a better outcome than surgery alone. Twelve cases developed recurrence. Compared to DCA, ACC had earlier staging and better survival. The overall 1-, 2-, and 5-year survival rates for patients with ACC were 73.7, 26.3, and 5 %, respectively.

**Conclusions:**

ACC carries a better prognosis than DCA and a similarly high recurrence rate, while surgical resection proved the best first-line approach for it. A well-planned neoadjuvant or adjuvant chemoradiotherapy indeed benefit the patients with ACC.

## Background

Acinar cell carcinoma (ACC) is a relatively rare pancreatic neoplasm with poorly defined prognosis [1, 2]. The first case of ACC was reported by Berner in 1908 [3]. He described a kind of syndrome that is characterized by fever, polyarthritis, subcutaneous fat nodular necrosis, and eosinophilia. Now it is known to be secondary to lipase hypersecretion by the tumor and recognized as lipase hypersecretion syndrome [1, 2, 4–6]. It is a kind of huge, exophytic, well-circumscribed, and hypovascular mass and favors a head of the pancreas distribution topographically, but sometimes in any other part of the pancreas.

The tumor is classically seen in older male, usually in their sixth or seventh decade. Although the pancreas is made up predominantly of acinar cells by 82 % in volume [7], the ACC accounts for approximately 1 % of all primary pancreatic neoplasm [8–10]. The reason is still unclear, and some researchers speculated that acinar cells may undergo a metaplasia into ductal cells when they met with genetic instability [11–15]. According to reports in the literature, the prognosis of ACC remains a controversy, mixed with a poorer prognosis [9, 16] and others showing a better prognosis compared to DCA [1, 2, 17]. In contrast, it is generally accepted that most patients with ACC have high rates of recurrence [1, 2, 18]. Furthermore, some estimated ACC to be more indolent, similar to the neuroendocrine pancreatic tumors [1]. Past research reported median survival for ACC ranging from 18 to 33 months [1, 2, 16, 18, 19], with a 39-patient single-institution case series demonstrating a 19-month median survival [2]. In general, the preoperative diagnosis of ACC is rarely achieved and the prognosis is poor, due to the metastatic disease and a high recurrence rate [1, 2].

There have been a limited number of small case series concentrating on the prognosis and clinical features of ACC [1, 2, 6, 16, 18–22]. Thus, the objective of this study is to present the experience of ACC to better understand its clinical characteristics, pathology, treatment, survival outcomes, and patterns of recurrence. We also determined that there was a significant difference in the survival of ACC compared to DCA.

## Methods

### Patients

We performed a retrospective review of prospectively collected surgical and pathological databases between 1995 and 2015 at Tianjin Medical University Cancer Institute and Hospital for all cases of acinar cell carcinoma of the pancreas, and 19 patients with pathologically confirmed ACC were enrolled, while 19 conventional DCAs assigned randomly as control. For the patients within the study, we collected their demographic information, preoperative clinical symptoms, imaging results, operative and pathological findings, tumor size and staging, postoperative complications, mortality, and survival. As part of our study, all the patients had tissue specimens from surgical resection or pathological results at our institution. The study was approved by the Institutional Review Board (IRB) of Tianjin Medical University Cancer Institute and Hospital. All patients provided written consent for the storage of their information in the hospital database and for the use of this information in our research.

### Follow-up and survival endpoints

Postoperative follow-up was performed through a hospital visit, by telephone, or by mail every 3 months. Follow-up was available for all 19 patients with a median follow-up time of 22 months. The endpoint of the study was overall survival (OS). OS was calculated as a period of time from the date of diagnosis to the date of death of any cause or the date of last follow-up.

### TNM staging of ACC and DCC

According to the 7th edition of the AJCC (American Joint Commission on Cancer) TNM staging system, postoperative T staging was as follows: T0, no evidence of primary; Tis, in situ; T1, limited to pancreas (≤2 cm); T2, limited to pancreas (>2 cm); T3, extends beyond pancreas, no involvement of CA or SMA; T4, involves CA or SMA. Postoperative N staging was as follows: N0, no nodal metastasis; N1, regional lymph node metastasis. Postoperative M staging was as follows: M0, no distant metastasis; M1, distant metastasis.

### Statistical analysis

Clinicopathological features and survival were analyzed. Mean and median survival were estimated using Kaplan-Meier methods and compared through the log-rank test. Potential prognostic factors were tested using the log-rank test. Categorical variables were compared using the chi-square test. For all tests, *P* values <0.05 were considered statistically significant.

## Results

### Patient characteristics

From 1995 to 2016, 19 patients pathologically proven ACC were identified in Tianjin Medical University Cancer Institute and Hospital from a retrospective review of the pathology database, assigned to the case group. For comparison to conventional ductal cell adenocarcinoma (DCA), we assigned 19 matched cases to the control group randomly during the same time (Table 1). The mean and median ages of patients in our institution were 54.2 and 54 years (range 39–77), respectively, with 84.2 % being male. Prevalent clinical symptoms were abdominal pain or discomfort (*n* = 16), weight loss (*n* = 19), back pain or discomfort (*n* = 14), pancreatic leakage (*n* = 10), bile leakage (*n* = 9), diabetes (*n* = 9), and nausea/vomiting (*n* = 7). Tumor location was predominantly in the head (*n* = 8) and tail (*n* = 9) and only a few in the body (*n* = 2). Different from the conventional ductal cell adenocarcinoma (DCA), no jaundice was found in patients with a head cancer, which was the classic presentation [10]. Furthermore, the tumor marker CA19-9 was commonly elevated in invasive DCA, and according to the laboratory examination, there was no elevated CA 19–9 with a median value of 21.70 U/L (range 4.6–28.1 U/L). Two patients (patient 4, 15) were tested to have elevated serum lipase, however, without classic clinical manifestations of lipase hypersecretion syndrome. Of the case group (*n* = 19), features seen on preoperative CT scan included a hypovascular-hypodense mass (*n* = 18), exophytic tendency (*n* = 14), well-circumscribed thickened border structure (*n* = 10), and necrosis within the tumor (*n* = 7).

**Table 1:** Patient characteristics

Patient	Age	Gender	Stage and T	Size (cm)	Operation	Survival (months)	Recurrence	Chemo/rad	Alive/dead
1	58	Male	IIAt3	5	Distal pancreatectomy	23	Local	+/−	Alive
2	54	Male	IBt2	4.2	Pancreaticoduodenectomy	76	None	+/−	Alive
3	46	Male	IIIt4	3.5	Distal pancreatectomy	11	Liver	−/−	Dead
4	41	Male	IBt2	3.7	Distal pancreatectomy	54	Local	+/−	Alive
5	52	Female	IVt4	5.7	None	5	Liver	−/−	Dead
6	55	Male	IIIt4^a^	7.6	Gastrojejunostomy	13	Liver	+/+	Dead
7	52	Male	IBt2	9	Pancreaticoduodenectomy	19	Local	+/−	Alive
8	39	Male	IIBt3	11.8	Pancreaticoduodenectomy	17	Local	−/−	Dead
9	47	Female	IIBt2	6.3	Pancreaticoduodenectomy	21	None	+/−	Alive
10	49	Female	IBt2	5.4	Distal pancreatectomy	34	None	+/−	Alive
11	54	Male	IIBt3	6.5	Pancreaticoduodenectomy^c^	16	Local	+/−	Dead
12	48	Male	IIAt3	2	Distal pancreatectomy	33	None	+/−	Alive
13	49	Male	IIBt2	7.6	Distal pancreatectomy	21	None	+/−	Alive
14	71	Male	IVt4	5.6	None	11	Distant	+/−	Dead
15	60	Male	IIAt3^b^	5.4	Distal pancreatectomy	21 days	None	−/−	Dead
16	56	Male	IIBt3	3.5	Pancreaticoduodenectomy	18	None	+/−	Alive
17	68	Male	IVt4	4.9	None	9	Local	+/−	Dead
18	77	Male	IIBt3	5.7	Distal pancreatectomy	17	Local	+/−	Alive
19	54	Male	IIAt3	2.8	Pancreaticoduodenectomy	27	Local	+/−	Alive

^a^Superior mesenteric artery (SMA) and port vein (PV) were found invaded by the tumor in initial operation, so the patient received a palliative gastrojejunostomy

^b^Patient was found suffering from an infection of biliary tract after pancreaticoduodenectomy

^c^Patient was found to be locally advanced during the initial exploratory laparotomy, then received neoadjuvant chemotherapy, and on re-exploration was found resectable

### Therapy procedure

**Fig. 1:**
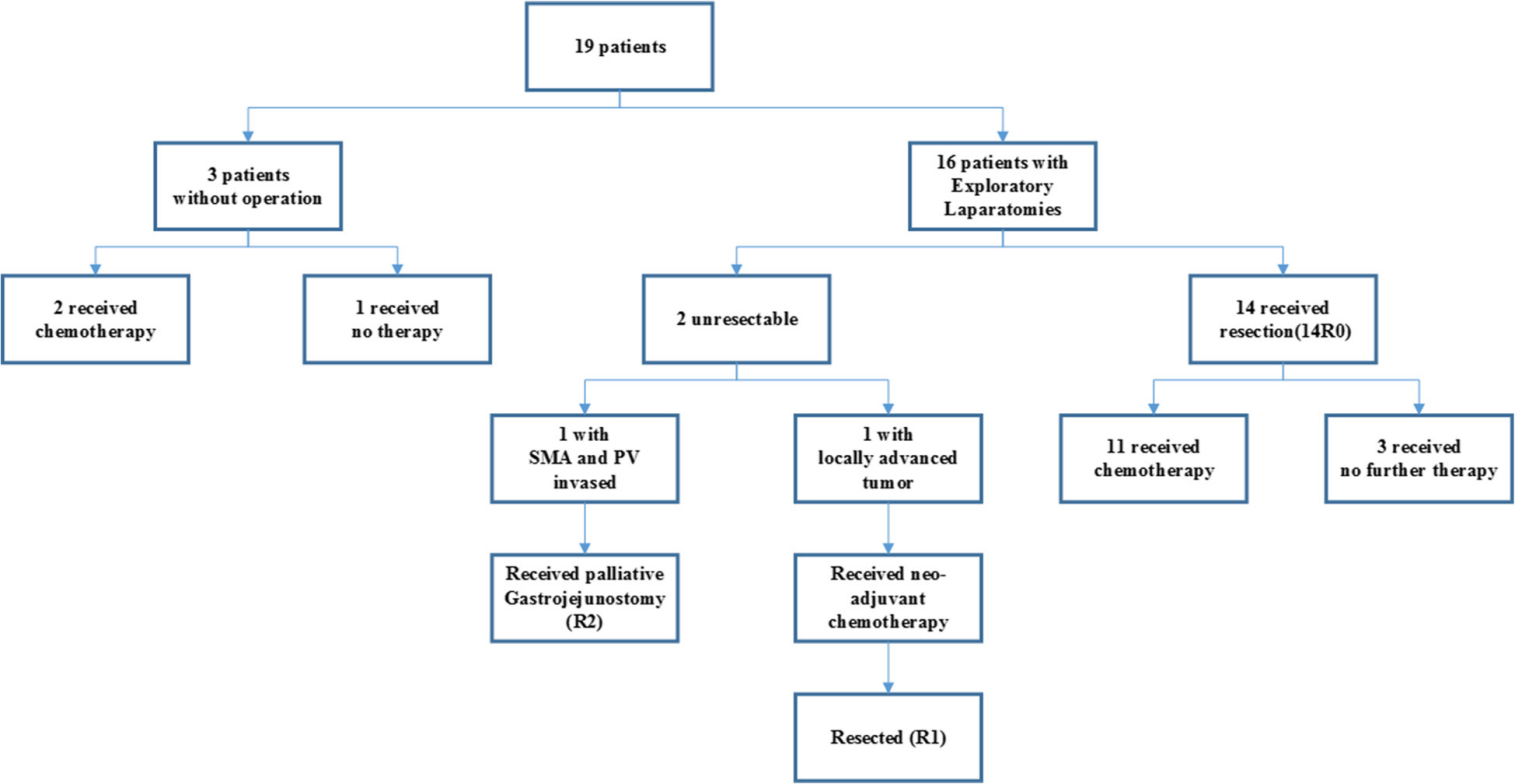
Schematic of patient treatment. Among 19 patients, three of them received palliative therapy because of the advanced diseases. The remaining 16 patients underwent exploratory laparotomy. Two of them were found unresectable, and the rest 14 patients received radical resection with R0 margins, 6 pancreaticoduodenectomy, and 8 pancreatectomy

Among these 19 patients in the case group (Fig. 1), three of them (patients 5, 14, and 17) were found extensive tumor invasion (SMA or transverse colon), metastatic disease (liver), or cancer cachexia during preoperative evaluation. None of them received radical or palliative resection. Two (patients 14 and 17) received chemotherapy and one (patient 5) received nothing for economic reasons. All the remaining 16 patients underwent exploratory laparotomy. During the initial operation, two of them (patients 6 and 11) were found unresectable. One (patient 11) with locally advanced tumor underwent subsequent neoadjuvant chemotherapy (gemcitabine) and was re-evaluated by CT scan, demonstrating a permission for surgical resection. The patient was resected (pancreaticoduodenectomy) at re-exploration with R1 margins. Another (patient 6) with SMA and PV invaded received palliative gastrojejunostomy with R2 margins and chemoradiotherapy postoperatively. The rest 14 patients underwent radical resection with R0 margins initially, with six patients undergoing pancreaticoduodenectomy and eight patients having a distal pancreatectomy with a concurrent splenectomy. Median survival was 18 month (range 1–76). Median resected tumor size was 5.40 cm (range 2.00–11.80). Among these resected cases, one of them (patient 15) was found suffering from an infection of biliary tract after pancreaticoduodenectomy and died 21 days postoperatively of septic shock. According to the 7th edition of the AJCC (American Joint Commission on Cancer) TNM staging system, the case group tumor pathological staging were stage I (*n* = 4), stage II (*n* = 10), stage III (*n* = 2), and stage IV (*n* = 3). Of the patients who underwent exploratory laparotomy (*n* = 16), all of them had postoperative pathology confirming a diagnosis of ACC. Of the remaining three patients, each of them was diagnosed by cytopathology from endoscopic retrograde cholangiopancreatography (ERCP) brushing. To a certain extent, further diagnostic immunohistochemical stains for antichymotrypsin and antitryptase (*n* = 10) also made great contributions to pathologic diagnosis of ACC. The lipase examination was done in all 19 patients, revealing positive results in patients 2, 4, 6, 14, and 17, respectively.

### Follow-up

**Fig. 2:**
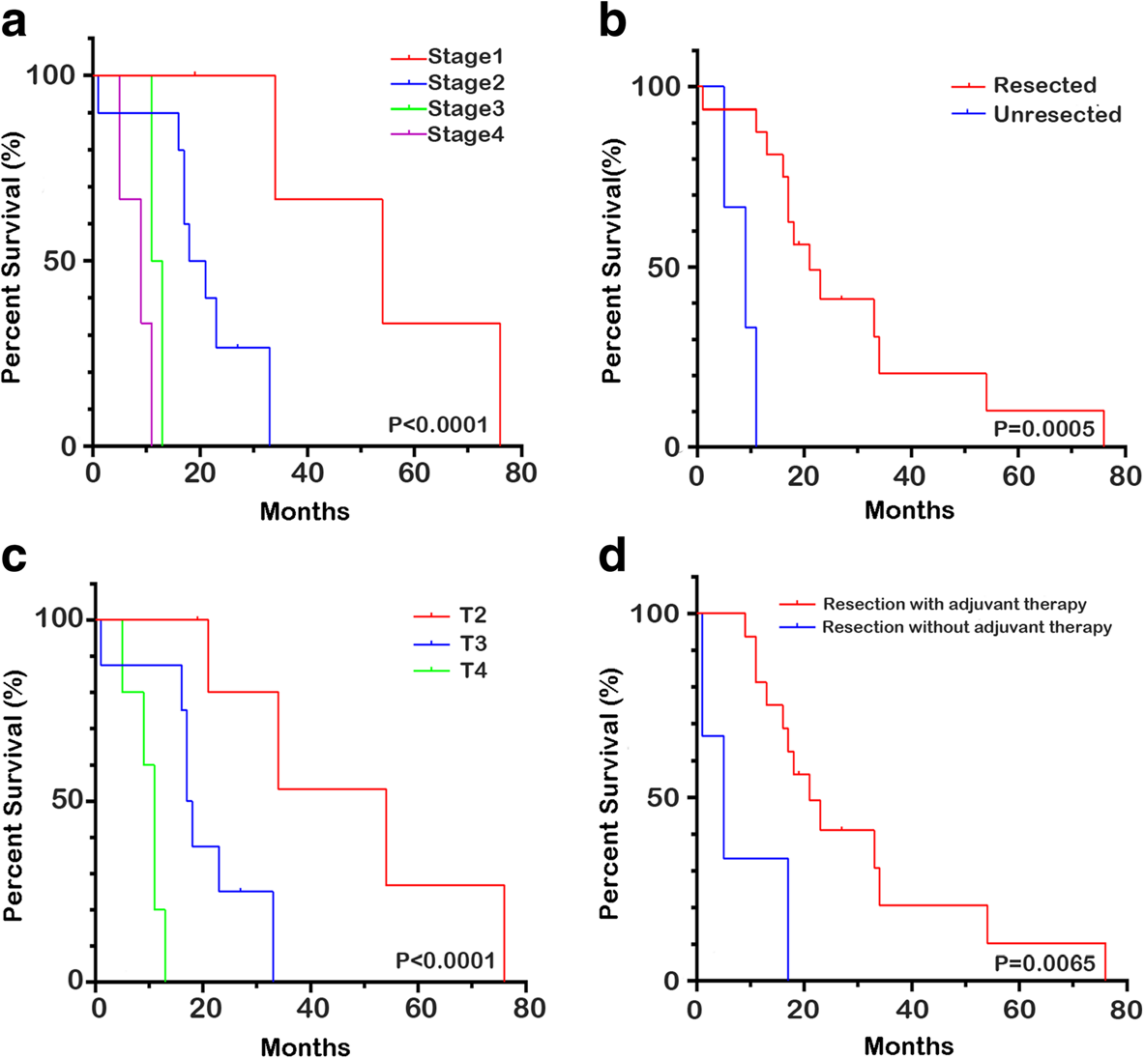
The Kaplan-Meier actuarial survival curves for ACC patients. **a** Overall stage-specific survival were stage I 21.0 % (mean survival 45.75 months), stage II 52.6 % (mean survival 19.4 months), stage III 10.5 % (mean survival 12.0 months), and stage IV 15.8 % (mean survival 8.33 months). **b** The resected cases had a significantly better survival than those without resection (median survival 19 vs. 9 months, *P* < 0.0001). **c** Among resected cases, earlier T classification was associated with a longer survival time (*P* < 0.05). **d** Resection followed by postoperative adjuvant therapy had a better outcome than surgery alone (*P* = 0.006)

Postoperative follow-up was available for all cases. Median survival time in the case group (*n* = 19) was 18 months, with actuarial survival at 1, 2, and 5 years estimated to be 73.7, 26.3, and 5 %, respectively (Table 2). Twelve cases (63.1 %) developed recurrence (8 local, 3 liver, 1 distant), demonstrated on postoperative follow-up CT scans (Table 1). Overall stage-specific (Fig. 2a) survival were stage I 21.0 % (mean survival 45.75 months), stage II 52.6 % (mean survival 19.4 months), stage III 10.5 % (mean survival 12.0 months), and stage IV 15.8 % (mean survival 8.33 months). Earlier staging was associated with better 5-year survival (*P* < 0.05). For ACC, the resected cases had a significantly better survival than those without resection (median survival 19 vs. 9 months, *P* < 0.0001 Fig. 2b), while earlier T classification related to a longer survival time (*P* < 0.05 Fig. 2c). In our study, resected cases with postoperative adjuvant therapy had a better outcome than those received surgery alone (*P* = 0.006 Fig. 2d).

**Table 2:** Patient characteristics compared with DCA

		Acinar cell carcinoma	Ductal cell carcinoma	*P* value
Number of patients		19	19	
Gender	Male	16	10	0.036
	Female	3	9	
Median age (year)		54 (39–77)	65 (35–86)	0.001
Median survival (month)		18 (11–27)	4 (3–12)	<0.0001
Median tumor size (cm)		5.4 (2–11.8)	3.1 (2.1–6.4)	<0.001
Stage	S I	4	1	0.021
	S II	10	4	
	S III	2	2	
	S IV	3	12	
Location within pancreas	Head	8	14	0.013
	Body	2	4	
	Tail	9	1

### Analysis of prognosis

According to multivariable analysis (Table 3), the case group (*n* = 19) was predominantly male, larger tumors, less nodal metastases, and more frequently located in tail than in the head of the pancreas. Potential prognostic factors for long-term survival are earlier T classification and negative nodal metastases (Table 4).

**Table 3:** Factors associated with ACC compared to DCA

		Odds ratio (95 % CI)	*P* value
Gender	Female	1.0 (referent)	
	Male	4.667 (1.121–19.434)	0.034
Tumor size	<2.0 cm	1.0 (referent)	
	2.1–4.0 cm	0.233 (0.063–0.870)	0.030
	>4.0 cm	11.200 (2.882–43.531)	<0.0001
Nodal metastases	N1	1.0 (referent)	
	N0	12.444 (2.891–53.562)	0.001
Location	Head	1.0 (referent)	
	Body	1.580 (1.022–2.604)	0.004
	Tail	5.850 (1.464–23.377)	0.012

Odds ratios >1.0 indicate a higher likelihood of ACC compared to DCA. Factors that were not significant in the model were age, tumor grade, and distant metastases

**Table 4:** Potential prognostic factors for long-term survival after the resection of ACC

	Hazard ratio (95 % CI)	*P* value
Nodal metastases	20.370 (2.242–185.104)	0.007
T classification	16.308 (2.078–127.976)	0.008

Hazard ratios >1.0 indicate a higher risk of death within 5 years. Potential prognostic factors for long-term survival are earlier T classification and negative nodal metastases. Factors not significant in the model include resection margins and size of the tumor

### Comparison to DCA

**Fig. 3:**
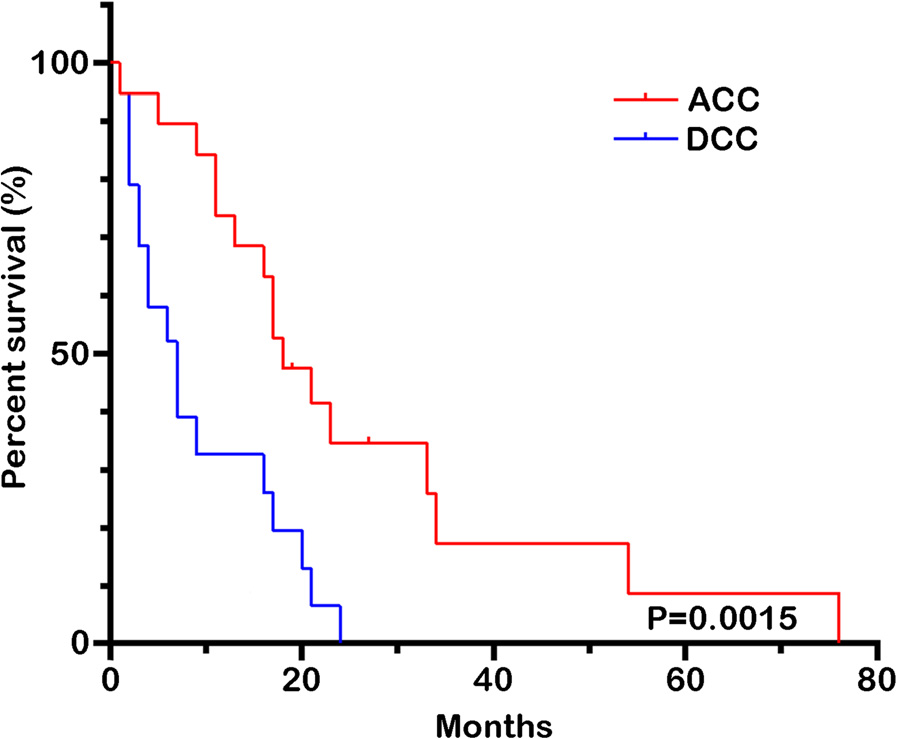
The Kaplan-Meier actuarial survival curves for the entire cohort by tumor type. The overall 1-, 2-, and 5-year survival rates for patients with ACC were 73.7, 26.3, and 5 %, respectively (median 18 months), whereas the overall survival rates for patients with DCA were 26.3, 2, and 0 %, respectively (median, 4 months, *P* < 0001)

For comparison to DCA, we assigned 19 matched cases to the control group randomly during the same time (Table 2). Compared with the control group (*n* = 19), the case group (*n* = 19) was younger (median 54 vs. 65 years, *P* = 0.001) and more likely to be male (84.2 % vs. 53.3 %, *P* = 0.027). Moreover, the case group had larger tumors (5.4 vs. 3.1 cm, *P* < 0.001), earlier staging (stage I + II 73.6 % vs. 26.7 %, *P* < 0.05), and longer survival time (18 vs. 4 months, *P* < 0.0001 Fig. 3). For the resected ones (Table 5), the case group (*n* = 16) had a larger male tendency than the control group (*n* = 14), and larger tumors (5.40 vs. 3.75 cm, *P* < 0.001). Based on the 7th edition of the AJCC criteria, ACC had earlier T classification (*P* = 0.023) and less nodal metastases (*P* = 0.001) than DCA.

**Table 5:** Tumor characteristics and treatments of resected patients

		ACC	DCC	*P* value
Number of patients		16	14	
Gender	Female	2	6	0.016
	Male	14	8	
Median tumor size (cm)		5.40 (2.00–11.80)	3.75 (2.1–6.4)	<0.001
T classification	T1	0	0	0.023
	T2	6	1	
	T3	8	7	
	T4	2	6	
Nodal metastases	N0	15	6	0.001
	N1	1	8	
Distant metastases	M0	14	12	0.919
	M1	2	2	
Location within pancreas	Head	7	10	0.004
	Body	1	3	
	Tail	8	1	
Margins	R0	14	11	0.929
	R1	1	2	
	R2	1	1	
Surgical procedure	Pancreaticoduodenectomy	7	9	0.008
	Distal pancreatectomy	8	1	
	Gastrojejunostomy	1	1	
	Total pancreatectomy	0	3	

## Discussion

Acinar cell carcinoma is a rare malignancy of the exocrine pancreas, and comprehensive retrospective institutional cases series are not easily available to make accurate conclusions of outcomes and clinical characteristics of ACC. This study, a total of 19 patients of ACC, aims to make contributions to the limited understanding about these lesions. By presenting experience from our institution, we sought to better define the clinical characteristics of ACC.

In our study, patients with ACC were predominantly males with median age of 54 years (range 39–77), younger than DCA. Holen et al. [2] and Klimstra et al. [1] also suggested an overall younger age of ACC than pancreatic adenocarcinoma and similar male/female ratio, 31:8 and 24:4, respectively. Meanwhile, ACC had larger tumors compared to DCA (5.4 vs. 3.1 cm, *P* < 0.001). Prevalent clinical symptoms in our study were abdominal pain or discomfort (*n* = 16), weight loss (*n* = 19), back pain or discomfort (*n* = 14), pancreatic leakage (*n* = 10), bile leakage (*n* = 9), diabetes (*n* = 9), and nausea/vomiting (*n* = 7), similar with the distribution commonly reported [23]. The classic presentation of painless obstructive jaundice did not occur in our study. Lipase hypersecretion syndrome is recognized to be secondary to lipase hypersecretion by the tumor, which is relatively specific for ACC [1, 2, 4–6]. Nevertheless, none of our patients presented with this syndrome confirmed the rarity reported by current literature [1, 2, 19].

During preoperative evaluation, features seen on CT scan included hypovascular-hypodense mass (*n* = 18), exophytic tendency (*n* = 14), well-circumscribed thickened border structure (*n* = 10), and necrosis within the tumor (*n* = 7). Consistent with our study, Tatli et al. reported ACC as an exophytic, well-circumscribed, hypovascular mass on CT scans, with necrosis when large [24]. Moreover, Chiou et al. described that ACCs were commonly hypodense mass on CT scans, accompanied with occasional enhancing capsules, calcification, intratumoral hemorrhage [19]. Although all of our patients were evaluated through abdominal CT preoperatively, none of them were definitively diagnosed through CT, emphasizing the nonspecificity to the diagnosis of ACC.

Although preoperative pathological diagnosis of ACC is rare, surgeon should keep ACC in mind when dealing with such kind of patients. ACC is reported a malignant tumor with poor outcomes [1, 2, 9, 16]; nevertheless, our study demonstrated that ACC was associated with better survival compared to DCA (median survival 18 vs. 4 months, *P* < 0.0001). Holen et al. [2] showed an overall median survival of 19 months. Similar median survival of 18.1 and 33 months were reported by Klimstra et al. [1] and Akhil K. Seth et al. [18], respectively, far better than the commonly reported median survival of DCA (6 months) [25]. Overall, patients with ACC had a better survival when compared to DCA [1, 2, 17]. However, we observed a high recurrence rate of 56.3 % in resected ones (9 of 16), including both local and distant metastases. Holen et al. [2] similarly reported recurrence rate of 72 %. Despite the well-circumscribed local confinement, ACC remains aggressive in nature, like other invasive pancreatic cancers, and is often a systemic disease with high recurrence rates [2]. Potential prognostic factors for long-term survival are earlier T classification and negative nodal metastases. Factors not significant in the model include resection margins and the size of the tumor.

Surgical resection remains the best first-line approach for ACC if lesion resection is possible. Holen et al. [2] reported a median survival of 36 months for resected ones, opposed to 14 months survival for those without any resection. Consistently, in our study, the resected patients had significantly better survival than the unresected ones (median 19 vs. 9 months, *P* < 0.0001). Therefore, surgical resection indeed makes a huge contribution to long-term survival in patients with ACC. Unfortunately, one of them (patient 15) was found suffering from infection of bile duct, as a postoperative complication, and died 21 days postoperatively of septic shock. In consequence, surgeon cannot be too careful to treat patients undergoing such kind of fetal operation, in case of deadly complications.

Whether ACC is sensitive to adjuvant therapy remains a controversial issue. According to a multivariable analysis reported by C. Max Schmidt et al. [12], patients with ACC had no better survival after adjuvant therapy. In contrast, a recent case report by Akhil K.Seth et al. [18], from Johns Hopkins, demonstrated that preoperative neoadjuvant therapy effectively downstaged four patients, using either gemcitabine, 5-fluorouracil, or adriamycin, ultimately allowing them as candidates again for surgical resection. Meanwhile, a multi-institutional series from J. M. Matos et al. [23] also contained patients downstaged by neoadjuvant therapy [23]. Our study also included a case (patient 11) which was effectively downstaged by neoadjuvant chemoradiotherapy. From our multivariate analysis, it indicated that receiving chemotherapy was associated with better OS (Table 2). These data, to some extent, are certainly encouraging that some patients appeared to benefit from this approach. Further study is required about the role of adjuvant and neoadjuvant therapy and whether they can improve survival of these patients. Due to endoscopic ultrasound-guided core biopsy and immunohistochemistry [26], the preoperative diagnosis of ACC is easier to achieve. Since aggressive surgery proved the most beneficial approach, patients with locally advanced or metastatic tumor should undergo well-planned neoadjuvant therapy, attempting for surgical resection.

Since such, the results above suggested that surgical resection remains the best first-line approach for ACC if lesion resection is possible due to its more favorable survival, and surgeon must be cautious with this aggressive malignancy, and guided their decision-making when faced with a potential ACC. Furthermore, to some extent, a well-planned neoadjuvant or adjuvant chemotherapy indeed benefit the patients. Nevertheless, limitations still exist during our research. Among these 19 ACC cases, some were admitted into institution many years ago, but detailed pathologic review was not available. Meanwhile, the sample size is relatively small. Consequently, further institutional and multi-institutional large scale studies are still required for a more detailed analysis of clinical manifestation, pathology, and appropriate treatment modalities of this rare malignant tumor.

## Conclusions

ACC carries a better prognosis than the more common DCA and a similarly high recurrence rate, while surgical resection proved the best first-line approach for it. Meanwhile, a well-planned neoadjuvant or adjuvant chemotherapy indeed benefit the patients with ACC.

## Abbreviations

ACC, acinar cell carcinoma; DCC, ductal cell adenocarcinoma
